# Acute Schistosomiasis in a Traveler Returning From St. Croix, United States Virgin Islands: A Case Report

**DOI:** 10.7759/cureus.105048

**Published:** 2026-03-11

**Authors:** John Weis, Mackenzie Caruthers, Megan Buller

**Affiliations:** 1 Internal Medicine, OhioHealth Riverside Methodist Hospital, Columbus, USA; 2 Infectious Diseases, OhioHealth, Columbus, USA

**Keywords:** eosinophilia, parasitology, returning traveler, schistosomiasis, travel medicine

## Abstract

In this case report, we describe a patient who presented to their primary care provider with abdominal pain after returning from travel to the Caribbean island of St. Croix. The patient reported two weeks of epigastric abdominal pain, dyspepsia, nausea, and night sweats. Following an initial evaluation that revealed eosinophilia with unclear etiology, they were referred to an infectious disease clinic. Through a targeted infectious evaluation and workup, the patient was diagnosed with acute schistosomiasis syndrome and was treated with praziquantel. Treatment resulted in the complete resolution of her symptoms, and follow-up laboratory evaluation showed complete resolution of her eosinophilia. This case highlights the need for ongoing surveillance and continued public health efforts to eliminate the disease, as well as the importance of a broad differential of peripheral eosinophilia in returning travelers.

## Introduction

The parasitic infection, schistosomiasis, is most commonly transmitted via contaminated freshwater sources through contact with skin. Schistosomiasis is most prevalent in Sub-Saharan Africa; however, it has been documented in other areas of the world, including the Caribbean. *Schistosoma mansoni *is the only documented schistosome species known to infect humans in the Caribbean region via its intermediate host, freshwater snails. Through public health efforts, previous studies have documented the disease’s epidemiological reign in the Caribbean. Today, it is well cited that Saint Lucia and Suriname are still considered endemic for schistosomiasis [1]. Public health efforts have eliminated the disease where it once flourished in the countries of Saint Martin, Saint Kitts, and Puerto Rico. There are also multiple countries in the Caribbean that do not have a known history of schistosomiasis. This includes the US Virgin Islands, where the patient returned from travel. The primary snail intermediate host of schistosomiasis in the Caribbean, *Biomphalaria glabrata*, is well documented on the island of Puerto Rico, but is not known to be endemic to the US Virgin Islands. However, a lesser-known snail intermediate host, *Biomphalaria helophila*, has been documented on St. Thomas, a neighbor to St. Croix [2].Focused eradication efforts of both snail host and the parasite are underway at the international level with assistance from the World Health Organization (WHO) and Pan American Health Organization (PAHO) [3]. These efforts include hydrological changes, the use of molluscicides, the addition of competitor snails, water and sanitation improvement, as well as health education. Nevertheless, cases of schistosomiasis are still arising, and according to a recent study, only 75% of treatment coverage was achieved in 12 reporting countries [4].

Although most commonly schistosomiasis is asymptomatic, it can manifest in acute and chronic syndromes, which can lead to significant morbidity and mortality. Cercarial dermatitis, more commonly known as swimmer’s itch, manifests as intense pruritus with urticarial rash shortly after swimming in fresh water in an endemic area. The other documented acute syndrome, acute schistosomiasis syndrome (Katayama fever), manifests in patients who have no prior exposure to schistosome species 2-12 weeks after exposure. Katayama fever can present in a variety of manifestations and, because of this, requires high clinical suspicion to make a diagnosis. Although most cases of Katayama fever may go undiagnosed and self-resolve, if the diagnosis is made, it is still recommended to be treated to prevent future complications from progression to chronic disease [5]. *S. mansoni* chronic infection is known to cause intestinal schistosomiasis, occurring greater than 12 weeks after exposure. Chronic intestinal schistosomiasis can progress to complicated disease, known as hepatosplenic schistosomiasis, and can lead to periportal fibrosis and the development of cirrhosis years after infection.

## Case presentation

A 59-year-old female was evaluated by their primary care physician for abdominal pain. The patient reported two weeks of dyspepsia, nausea, and night sweats. The review system was notable for the patient denied without associated weight loss, diarrhea, or constipation. Physical examination was notable for mild epigastric tenderness, with no rebound tenderness or guarding. Skin exam was negative for rash, erythema, or jaundice. The rest of the complete physical exam was unremarkable, and the patient’s vital signs were within normal limits, including a heart rate of 82 beats per minute (reference range 60-100 beats per minute). Complete blood count with differential showed a new abnormality of eosinophilia of 64% (reference range 1-4%) with an absolute count of 12.52 K/µL (reference range 15-500 cells/µL). Liver and kidney function were obtained with a complete metabolic panel, and all lab values were within normal limits. Outpatient computed tomography of the abdomen and pelvis with contrast was unremarkable for acute or chronic processes, with particular attention noted to the liver, pancreas, and kidneys, all noted to be normal.

With the new onset of eosinophilia, the patient was referred to an Infectious Diseases consultant. The patient's family and past medical history were reviewed and were unremarkable. Notably, there was no family history of any gastrointestinal malignancy, and the patient had a recent colonoscopy in the past year that was unremarkable. She lived in a rural Appalachian region of the United States, and upon further investigation into the patient's presentation, it was noted that she had recently returned from the Caribbean United States' Virgin Island of St. Croix one month prior to her onset of symptoms. While abroad, the patient ate local cooked fish, went snorkeling in the ocean, and walked barefoot on the beach without any known freshwater exposure. Following this detailed history, a broad differential diagnosis, including infectious and non-infectious etiologies, was developed. Given that the patient resided in Appalachia, a *Strongyloides *IgG antibody test was performed, which returned negative. With concern for inflammatory conditions with eosinophilia, such as eosinophilic granulomatous polyangiitis and hypereosinophilic syndrome, rheumatological serologies were ordered, including anti-nuclear antigen and anti-neutrophilic cytoplasmic antibody, which were also negative. The patient was negative for human immunodeficiency virus antibodies 1 and 2. IgG, IgM, and IgA were all within normal limits. IgE was noted to be elevated at 165 IU/mL (reference range 0.0-100 IU/mL). Because the patient had recently returned from travel in the Caribbean and schistosomiasis is known to be prevalent in neighboring Caribbean islands, a *Schistosoma *IgG antibody enzyme immunoassay (EIA) was performed, which returned positive (negative reference range). Other testing modalities, such as schistosomiasis stool antigen, PCR, and ova and parasite testing, were not obtained due to laboratory limitations. A list of relevant laboratory values obtained is summarized in Table 1.

**Table 1:** Laboratory Values WBC: white blood cells; Abs: absolute; AST: aspartate aminotransferase; ALT: alanine aminotransferase; ANA: antinuclear antibody; Ig: immunoglobulin; IgE: immunoglobulin E; IgA: immunoglobulin A; IgG: immunoglobulin G; IgM: immunoglobulin M; HIV: human immunodeficiency virus; P-ANCA: perinuclear anti-neutrophil cytoplasmic antibody; C-ANCA: cytoplasmic anti-neutrophil cytoplasmic antibody

Parameter	Patient Value	Reference Range	Units
WBC	20.38	4.50-11.00	K/mcL
Neutrophils	15.10	40.00-60.00	%
Lymphocytes	15.30	20.00-40.00	%
Monocytes	4.70	2.00-8.00	%
Eosinophils	63.70	1.00-4.00	%
Basophils	0.90	0.50-1.00	%
Neutrophils Abs	3.00	1.70-7.00	K/mcL
Lymphocytes Abs	3.00	0.90-4.00	K/mcL
Monocytes Abs	0.92	0.30-0.90	K/mcL
Eosinophils Abs	12.52	0.00-0.50	K/mcL
Basophils Abs	0.17	0.00-0.30	K/mcL
Sodium	136	135-145	mmol/L
Potassium	4.1	3.50-5.10	mmol/L
Creatine	0.89	0.40-1.10	mg/dL
AST	18	0.00-45	U/L
ALT	28	40-159	U/L
Alkaline Phosphatase	86	14-65	U/L
Total Bilirubin	0.3	0.00-1.30	mg/dL
*Strongyloides* Antibody, IgG	negative	negative	-
*Schistosoma* Antibody, IgG	positive	negative	-
P-ANCA	negative	negative	-
C-ANCA	negative	negative	-
ANA	<1:80	<1:80	-
HIV antibody	negative	negative	-
IgE	165	0.00-100.00	IU/mL
IgA	217	84-381	mg/dL
IgG	1,029	541-1694	mg/dL
IgM	69	43-238	mg/dL
Lipase	101	73-393	U/L

The patient was subsequently diagnosed with acute schistosomiasis syndrome and treated with a single 2,400 mg dose of praziquantel. Repeat blood count with differential at one-month follow-up resulted in resolution of the eosinophilia as well as resolution of her symptoms. She was treated with an additional 2,400 mg dose of praziquantel six weeks following the first dose, and at subsequent follow-up visits with her primary care physician, the patient reported no recurrence of her initial symptoms, including abdominal pain, dyspepsia, or night sweats. Particularly, she has not had any recurrence of abdominal pain, dyspepsia, or night sweats.

## Discussion

The diagnosis of acute schistosomiasis syndrome is most often established clinically in patients with a relevant epidemiological history and compatible clinical symptoms, supported by absolute eosinophilia with a count greater than 7.5 K/µL. Currently, microscopic examination of stool and urine is the gold standard diagnosis of schistosomiasis. *S. mansoni* is typically an intestinal schistosome, and the Kato-Katz fecal smear is the gold standard microscopic evaluation of this organism [6]. However, this diagnostic option is often limited due to laboratory availability and experience. Serologic testing is a useful testing modality in individuals who reside in areas that are not endemic to schistosomiasis. Careful interpretation of schistosomiasis serology is needed as this antibody often stays positive for life, and itself is not sufficient alone to make a diagnosis of acute schistosomiasis syndrome without relevant clinical history.

Our patient had no previous travel to endemic regions, had no documentation of previous eosinophilia, and her non-specific abdominal pain, cough, and nausea are suggestive of the syndrome. With ambiguous clinical manifestations and often difficult interpretation of laboratory testing, there are no guidelines and a lack of a standardized consensus from world organizations on the case definition of schistosomiasis. With this, many cases are not identified or misdiagnosed. A recent Delphi consensus study reported that a confirmed case of acute schistosomiasis can be defined as a person with relevant epidemiological history, possible infective contact from three weeks to three months, with or without acute schistosomiasis syndrome symptoms, and the presence of eggs in biological material, positive PCR, positive circulating anodic antigen (CAA) antigen, or documented seroconversion [7]. This definition supports our patient as a confirmed acute schistosomiasis syndrome case as her case fulfilled the criteria with relevant epidemiological history, infective contact within the prior one month, clinical symptoms, and documented seroconversion with positive *Schistosoma* IgG antibody. With an often-ambiguous diagnosis, there is also a lack of guideline-directed treatment recommendations [8]. The pathophysiology of the Katayama fever is suspected to correlate with the initiation of egg production and a steep rise in antigen burden. There is some data that suggests that patients should receive glucocorticoids to reduce inflammation, particularly in early diseases, at less than 12 weeks. The current standard of care has no comment on dose, route, or duration of corticosteroid administration, especially in patients with mild to moderate disease severity. In patients with severe disease, case series have shown improvement with methylprednisolone 0.5 mg/kg recommended two hours following the dose of praziquantel as the glucocorticoids reduce plasma levels of the anti-parasitic agent [9]. It is generally recommended that an additional dose of praziquantel be administered weeks after the first treatment to eliminate remaining schistosomes that matured since the original treatment; however, the timing of repeat treatment has not been well studied. There are no current guidelines on lab monitoring following treatment. Although eosinophilia is noted as part of the diagnostic criteria for acute schistosomiasis syndrome, it is recommended that if eosinophils remain increased after three months, this may indicate treatment failure or an alternative etiology requiring further investigation by healthcare providers [10].

## Conclusions

Eosinophilia in returning travelers from the Caribbean should raise concern for the neglected tropical disease of *S. mansoni*. The prevalence of *S. mansoni* in St. Croix is currently unknown. To the best of our knowledge, the case described above represents the first reported case of acute infection with *Schistosoma* documented following travel to the island. This case highlights the need for future studies to evaluate the prevalence and meet the goals set by the WHO to eradicate the disease, as it continues to lead to significant morbidity, particularly among children in developing countries. As in this case, primary care physicians are often the first healthcare providers to encounter returning travelers and should be aware of the disease manifestations of schistosomiasis in travelers returning from tropical regions to identify early detection of the organisms. Informed consent for this publication was obtained via telephone; the patient continues to have no recurrence of her symptoms from the initial presentation.
